# Differing Trade-Off Patterns of Tree Vegetative Organs in a Tropical Cloud Forest

**DOI:** 10.3389/fpls.2021.680379

**Published:** 2021-07-21

**Authors:** Yuanyuan Yang, Chuchu Xiao, Xianming Wu, Wenxing Long, Guang Feng, Guoying Liu

**Affiliations:** ^1^College of Ecology and Environment, Hainan University, Haikou, China; ^2^National Positioning Observation and Research Station of Forest Ecosystem, College of Forestry, Hainan University, Haikou, China; ^3^Key Laboratory of Tropical Forest Flower Genetics and Germplasm Innovation, Ministry of Education, Haikou, China; ^4^Bawangling Branch, Hainan Tropical Rainforest National Park Administration, Changjiang, China

**Keywords:** plant economic spectrum, resource use strategy, nutrient traits, morphological trait, above- and below-ground correlations

## Abstract

Functional trait ecology demonstrates the significance of the leaf economics spectrum in understanding plants’ trade-off between acquisitive and conservative resource utilization. However, whether trait variations of different vegetative organs are coordinated and whether the plant economics spectrum is characterized by more than one vegetative organ remain controversial. To gain insights into these questions, within a tropical cloud forest in Hainan Island, a total of 13 functional traits of 84 tree species were analyzed here, including leaf, stem and root traits. By using standardized major axis (SMA) regression and principal components analysis, we examined the trait variations and correlations for deciphering plants’ trade-off pattern. We found decreases of leaf phosphorus content, leaf nitrogen content and specific leaf area and increases of leaf mass per unit area (LMA), wood density and leaf thickness along the first principal component, while there were decreases of specific root length and specific root area and increases of root tissue density along the second principal component. Root phosphorus and nitrogen contents were significantly positively associated with the phosphorus and nitrogen contents of both stem and leaf. Wood density was significantly positively associated with LMA and leaf thickness, but negatively associated with leaf thickness and specific leaf area. Our results indicate that, in the tropical cloud forest, there is a “fast–slow” economic spectrum characterized by leaf and stem. Changes of nutrient trait are coordinated, whereas the relationships of morphological traits varied independently between plant above- and below-ground parts, while root nutrient traits are decoupled from root morphological traits. Our findings can provide an insight into the species coexistence and community assembly in high-altitude tropical forests.

## Introduction

Plant functional traits individually or jointly indicate species ecological functions and ecosystem response to environmental changes (Cornelissen et al., 2003). Thus, the functional trait approach has been used as a tool to comprehend the spectrum of plant functional strategies and their relationships with the environment (Westoby and Wright, 2006; Garnier and Navas, 2012). In the Plant Ecology Strategy Schemes (PESS), species are arranged in several spectra according to category and ecological attribute, representing the multiple trade-offs of resource investment (to cells, tissues, and organs) among species (Grime, 1977; Westoby, 1998; Westoby et al., 2002; Diaz et al., 2004; Freschet et al., 2010).

As a response to the local environmental conditions, different plant organs often develop in coordination with each other, alternating the resource utilization strategies to maintain growth and development (Reich et al., 1997; Ryser and Eek, 2000; Poorter et al., 2012). For example, the leaf economic spectrum (LES) revealed by Wright shows that the variation and correlation of leaf traits reflect an ecological trade-off in resource utilization, which has been widely confirmed by ecologists (Reich et al., 1998; Enquist et al., 1999; Santiago et al., 2004; Poorter et al., 2008; Cornwell and Ackerly, 2009; Baraloto et al., 2010). However, it remains controversial whether the stems and roots also display such a one-dimensional strategic trade-off (Freschet et al., 2010; Prieto et al., 2015). Reich and Cornelissen (2014) reveal a one-dimensional trade-off among traits related to resource acquisition and storage in fine roots (i.e., a root economic spectrum, RES), caused by selection pressure of the external environmental and biophysical constraints. The multi-dimensional hypothesis of roots, on the contrary, states that there is no one-dimensional trade-off among root traits (Kramer-Walter et al., 2016; Weemstra et al., 2016). Such a reality suggests that more attempts should be done for testing whether the trade-off is one-dimensional.

Moreover, for plants, it is still unclear whether there is a coordinated trait change between above- and below-ground organs (in response to e.g., environmental change). The morphological similarity hypothesis holds that because there is anatomical continuity between the xylem and phloem tissues between the root and stem systems (Tyree and Ewers, 1991; Pratt et al., 2007), and that the root morphological traits (e.g., wood density) will coordinate with the stem morphological traits. But the functional similarity hypothesis states that root traits closely coordinate with leaf traits, rather than with stem traits, because the leaf function depends on the water and nutrients absorbed by the roots, and the root growth in turn depends on the carbohydrates produced by the leaves (Chapin, 1980; Poorter et al., 2008; Baraloto et al., 2010). Recent studies have found that leaves and roots evolve independently to adapt to environmental conditions (Kramer-Walter et al., 2016; Weemstra et al., 2016), resulting in a multi-dimensional trade-off. Such a controversy suggests that more studies are needed to test the correlations between above- and below-ground traits.

Tropical cloud forests are mainly distributed in tropical parts of the Americas, Africa, and Asia (Stadtmüller, 1987; Bubb et al., 2004). Compared with low-altitude tropical forests, tropical cloud forests are characterized by low temperature, strong winds, high frequency of clouds and fog, low soil phosphorus, and intense ultraviolet radiation (Long et al., 2011a). Trees in the tropical cloud forest usually have curved trunks, with relatively small height and diameter, and high density. They also have compact canopies and small leathery and stiff leaves with a higher specific leaf area (SLA; Long et al., 2011b). A recent study further found that there was a positive correlation between the leaf mass per unit area (LMA), plant height (H), and wood density (WD) of tropical cloud forest trees (Long et al., 2020), indicating great carbon investment differences in leaf-stem and stem height-stem density. However, it is not clear whether plants in the tropical cloud forest also possess a plant economic spectrum (PES) for all vegetative organs, and how plants balance and optimize resource allocation among roots, stems, and leaves.

To address these issues, we measured 13 functional traits (Table 1) of 84 tree species in the Bawangling tropical cloud forest, Hainan Island, and analyzed their variations and correlations by using multivariate and bivariate analyses. Specifically, we measured the leaf traits related to resource acquisition and defense (Wright et al., 2004; Agrawal and Fishbein, 2006), the root traits related to nutrient absorption, transportation, and defense, and the stem traits related to nutrient transportation and defense (Pratt et al., 2007; Chave et al., 2009). We tested the following two hypotheses in the tropical cloud forest: (1) tropical cloud forest tree roots, stems, and leaves would show a PES to balance and optimize resource allocation, and that (2) the resource investment between above- and belowground traits of vegetative organs would be coordinated.

**TABLE 1:** List of the 13 functional traits, as well as their ecological strategies.

Organ	Trait	Abbreviation	Unit	Strategy
Fine root	Specific root length	SRL	cm g^–1^	Resource capture
	Specific root area	SRA	mm^2^ mg^–1^	Resource capture and defense
	Root tissue density	RTD	g cm^–3^	Transport, structure, and defense
	Root nitrogen content	RN	%	Resource capture and defense
	Root phosphorus content	RP	%	Resource capture
Leaf	Leaf thickness	LT	cm	Resource capture and defense
	Specific leaf area	SLA	mm^2^ g^–1^	Resource capture and defense
	Leaf mass per unit area	LMA	g cm^–2^	Resource capture and defense
	Leaf nitrogen content	LN	%	Resource capture and defense
	Leaf phosphorus content	LP	%	Resource capture
Stem	Wood density	WD	g cm^–3^	Transport, structure, and defense
	Stem nitrogen content	WN	%	Resource capture and defense
	Stem phosphorus content	WP	%	Resource capture

## Materials and Methods

### Site Conditions

This study was conducted in the tropical cloud forest of Bawangling Mountain (19°04′57.93″ N, 109°12′13.93″ E), Hainan Island, south China. Tropical cloud forest in Hainan Island is composed of primary growth forest (no history of human disturbance) ca. 0.40 km^2^ in area, mainly distributed as mountaintop islands starting above altitudes of 1,250 m. The mean daily air temperature in the rainy season (May–Oct.) ranges from 17.6 to 24.8°C, and the mean daily relative humidity in the rainy season ranges from 87.88 to 100%; Specifically, the mean daily air temperature in the dry season (Nov–Apr.) ranges from 15.2 to 22.6°C, and the mean daily relative humidity in the rainy season ranges from 57.68 to 100% (Long et al., 2011a). The main soil types are lateritic soil and mountainous red soil. The study forest is located on an eastern slope located on the steep slopes (36–45°). Average tree height is 4.8 ± 2.8 m, and tree densities were 9,633 stems ha^–1^ [for all trees with dbh (diameter at breast height) ≥ 1 cm] (Long et al., 2011a). Dominant tree species include *Pinus fenzeliana*, *Distylium racemosum, Syzygium buxifolium*, *Engelhardia roxburghiana*, and *Rhododendron moulmainense*.

### Data Collection

Twenty-one 20 m × 20 m plots were established in the Bawangling tropical cloud forest, with more than 50 m distance between adjacent fields. Each 400 m^2^ field was divided into four 10 × 10 m subplots and 16 5 × 5 m quadrats using the neighbor grid method. We record the species, height and DBH (diameter at breast height) of all individual trees appearing in the study plots, with dbh ≥5 cm using a clinometer. A total of 13 root, stem, and leaf functional traits, related to plant resource strategies, were determined for three trees of each species in the field (Table 1).

To determine leaf traits, we collected and measured 2–3 recently expanded sun leaves (current year’s growth) from each individual for three standard trees of each species. The leaf thickness was measured with digital display vernier caliper (SF2000,Guilin, China). Leaf area was quantified using a leaf area meter (LI-COR 3100C Area Meter, LI-COR, United States). Leaves were then dried to a constant weight at 70°C for at least 72 h and weighed by electronic balance (AR2140, OHAUS, United States); leaf area and dry mass were then used to calculate LMA (mg mm^–2^) and specific leaf area (mm^2^ mg^–1^) for each tree.

To characterize species WD (g cm^–3^), we sampled three branches (1 cm ≤ dbh ≤ 2 cm) from each corresponding individual that was sampled for leaf traits. We removed the pith, phloem and bark, measured fresh volume on the rest of the branch using water displacement and determined dry mass after drying for 72 h at 70°C (Cornwell et al., 2006). Branch density is the dry mass of the rest of the branch (minus the pith, phloem, and bark) divided by its volume, and has been demonstrated to be closely related to core stem density for adult trees in BNR (*R*^2^ = 0.93; Bu et al., 2014). Therefore, the branch density can be used to represent the wood density, so as to avoid the damage by using the growth cone drill to get the tree rings.

For fine root sampling, following the same procedure as in Guo et al. (2008), three root samples for each species (one sample from each of the three chosen trees) were collected from 0 to 20 cm of soil. A 1 × 1 m subplot was first identified within a 2-m distance of the tree stem. Then a fork was used to loosen the soil in the sampling area. Root branches were followed to the tree stem and cut from the main lateral woody roots. Roots were then rinsed, placed onto a mesh tray filled with distilled water and spread out to avoid any overlap between roots. The absorption roots (diameter < 2 mm) were scanned by EPSON EXPRESSION 700XL colored scanner (dots per inch = 400). The scanned images of each segment allowed us to measure root morphological traits using WinRHIZO Pro 2012 b software. All scanned roots were then oven-dried for 72 h at 80°C and weighed to determine their root dry matter. And then calculated the Specific Root Length (SRL, cm g^–1^), Root Tissue Density (RTD, g cm^–3^), and Specific Root Surface Area (SRA, cm^2^ g^–1^).

The nitrogen content was determined by Kjeldahl method, and the phosphorus content was decomposed by HClO_4_-H_2_SO_4_ digestion method, and then measured by Key-blue colorimetry.

### Data Analysis

The average values of 13 functional traits for each of the 84 species occurring in the 21 plots were calculated (Table 1). Multivariate and bivariate analyses were used, as a complement to each other, for assessing trait variations and correlations. Specifically, for each of the 13 functional traits, the average values were measured at the species level, and then a principal component analysis (PCA) was performed on these data (a matrix with 13 traits × 84 species), for evaluating the multivariate trait correlations among species, and for revealing the dimensionality of trait variations. The missing values in the dataset were processed with the missMDA package prior to PCA. To further examine the statistical significance of trait correlations, we performed pairwise Spearman correlation tests. For functional traits with significant correlations (*p* < 0.05), their bivariate relationships were carefully assessed using a SMA regression. It is known as a proper statistical technique for assessing the bivariate relationships without clear direction of causality (Warton et al., 2006). All data analyses were performed in R v4.0.2.

## Results

### PCA of Plant Functional Traits

**FIGURE 1:**
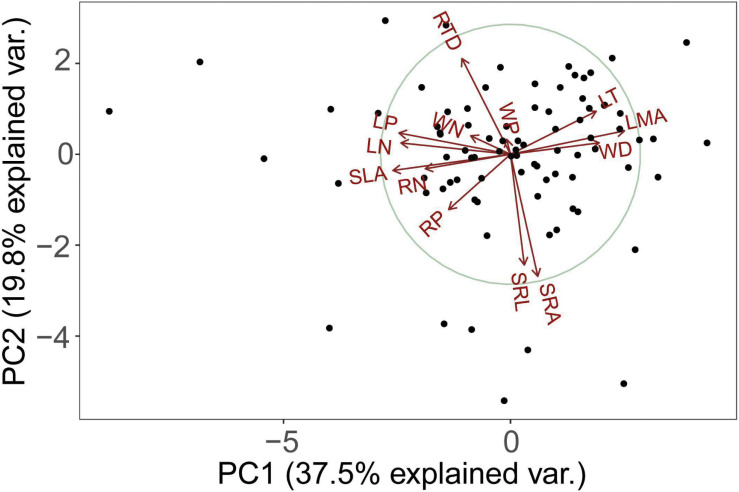
Principal component analysis (PCA) using average trait values from 84 tree species in the tropical cloud forest. See Table 1 for trait abbreviations.

The PCA analysis of 13 functional traits showed that root morphological traits (e.g., RTD, SRL, SRA) is orthogonal to leaf and stem morphological and chemical traits (Figure 1). The first two principal components explained 57.3% of the total variance (Figure 1). SLA, LN, RN, WN, LP, RP, and WP decreased but LT, LMA, and WD increased along this axis. In other words, one end of this axis is characterized by high LT, LMA, and WD, while another end is characterized by low LT, LMA, and WD. In one extreme (negative values) with high values of traits (SLA, nitrogen, and phosphorus content) that were positively correlated among themselves (Table 2) and representative of the resource acquisition strategy. At the opposite extreme (positive values) were species with high LT, LMA, and WD, these traits were also positively correlated among themselves (Table 2) and indicative of the resource conservation strategy.

**TABLE 2:** Spearman correlation coefficients of 13 plant functional traits within different components.

	SRL	RTD	SRA	RN	RP	LN	LP	WN	WP	SLA	LMA	LT	WD
SRL	1												
RTD	−0.46** *P* < 0.01	1											
SRA	0.88** *P* < 0.01	−0.78** *P* < 0.01	1										
RN	−0.23 *P* = 0.1	0.12 *P* = 0.4	−0.02 *P* = 0.15	1									
RP	0.03 *P* = 0.82	0.02 *P* = 0.9	0.01 *P* = 0.93	0.58** *P* < 0.01	1								
LN	−0.14 *P* = 0.33	0.24 *P* = 0.1	−0.24 *P* = 0.09	0.36* *P* = 0.01	0.32* *P* = **0.01**	1							
LP	−0.21 *P* = 0.15	0.23 *P* = 0.12	−0.23 *P* = 0.11	0.32* *P* = **0.01**	0.38** *P* < 0.01	0.68** *P* < 0.01	1						
WN	−0.21 *P* = 0.12	0.18 *P* = 0.18	−0.2 *P* = 0.15	0.27* *P* = **0.03**	0.29* *P* = **0.02**	0.07 *P* = 0.6	0.11 *P* = 0.39	1					
WP	−0.03 *P* = 0.83	0.16 *P* = 0.24	−0.03 *P* = 0.83	0.13 *P* = 0.31	0.39** *P* < 0.01	0.07 *P* = 0.61	0.08 *P* = 0.57	0.42** *P* < 0.01	1				
SLA	−0.1 *P* = 0.49	0.08 *P* = 0.55	−0.13 *P* = 0.33	0.17 *P* = 0.19	0.19 *P* = 0.15	0.5** *P* < 0.01	0.51** *P* < 0.01	0.3* *P* = **0.02**	−0.05 *P* = 0.72	1			
LMA	0.09 *P* = 0.52	−0.08 *P* = 0.54	0.12 *P* = 0.37	−0.22 *P* = 0.1	−0.23 *P* = 0.08	−0.57** *P* < 0.01	−0.58** *P* < 0.01	−0.28* *P* = **0.03**	0.05 *P* = 0.71	−0.97** *P* < 0.01	1		
LT	−0.06 *P* = 0.62	−0.06 *P* = 0.66	−0.04 *P* = 0.74	−011 *P* = 0.41	−0.11 *P* = 0.42	−0.39** *P* < 0.01	−0.36* *P* = 0.01	−0.11 *P* = 0.39	0.01 *P* = 0.95	−0.64** *P* < 0.01	0.7** *P* < 0.01	1	
WD	0.05 *P* = 0.7	−0.03 *P* = 0.79	0.07 *P* = 0.61	−0.18 *P* = 0.17	−0.31 *P* = 0.02	−0.36* *P* = **0.01**	−0.48** *P* < 0.01	−0.2 *P* = 0.11	0.02 *P* = 0.89	−0.5** *P* < 0.01	0.57** *P* < 0.01	0.53** *P* < 0.01	1

Significance values are in bold (*P < 0.05; **P < 0.01).

The second axis of the PCA is defined by the covariance between SRL and SRA, and RTD (Figure 1). SRL was significantly negatively correlated with RTD (Table 2) while significantly positively correlated with SRA.

### Bivariate Relationship Among the Root, Stem, and Leaf Traits

**FIGURE 2:**
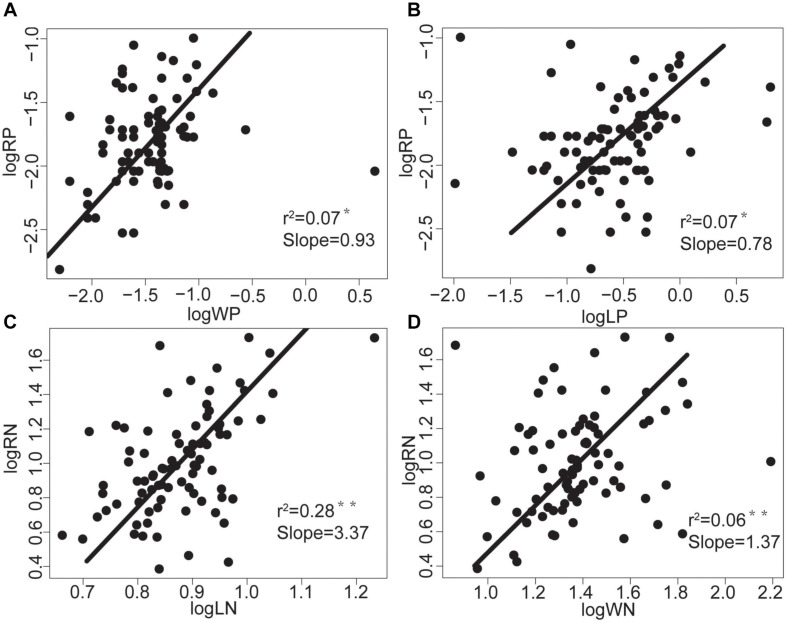
The relationships between nutrient functional traits (N, P concentration) of tree species’ above- and belowground vegetative organs in the tropical cloud forest. The slope values and *P*-values for **(A)** RP-WP, **(B)** RP-LP, **(C)** RN-LN, and **(D)** RN-WN derived from SMA analyses. * and ** indicate significant correlations at the levels of *P* < 0.05 and *P* < 0.01, respectively.

There were no significant correlations between the morphological traits of fine roots and those of stems or leaves (Table 2). However, RN was positively correlated with WN and LN (*n* = 84; *r*^2^ = 0.06, *P* < 0.05; *r*^2^ = 0.28, *P* < 0.01; Figure 2), and RP was positively correlated with WP and LP (*n* = 84; *r*^2^ = 0.07, *P* < 0.05; *r*^2^ = 0.07, *P* < 0.05; Figure 2).

**FIGURE 3:**
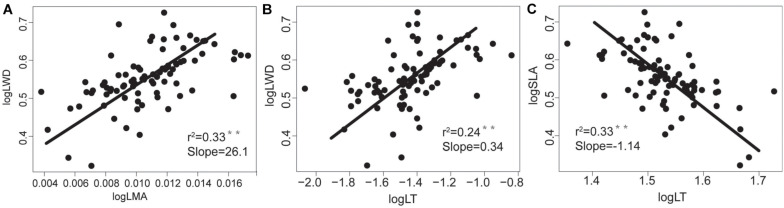
The relationships between morphological traits of tree species’ aboveground and underground organs in the tropical cloud forest. The slope values and *P*-values for **(A)** WD-LMA, **(B)** WD-LT and **(C)** SLA-LT derived from SMA analyses. * and ** indicate significant correlations at the levels of *P* < 0.05 and *P* < 0.01, respectively.

WD was negatively correlated with LN, LP, and SLA, and positively correlated with LT and LMA (Table 2 and Figure 2), indicating that the higher the WD, the greater the LMA and LT. In terms of nutrient traits, WN was positively correlated with LN and LP, while WP were not significantly correlated with the leaf traits (Table 2). The slopes with a significant correlation between different organs showed significant deviations from 1 (Figures 2, 3).

## Discussion

### Evidence of the “Leaf and Stem Economics Spectrum” in a Tropical Cloud Forest

The LES has been intensively studied in previous studies (Wright et al., 2004; Baraloto et al., 2010; Freschet et al., 2010), while whether PES only exists in leaf remains controversial (Baraloto et al., 2010; Freschet et al., 2010; Reich and Cornelissen, 2014; Prieto et al., 2015). By analyzing leaf, stem, and root trait variations and correlations in a tropical cloud forest, we found that the stems and leaves of trees jointly developed a fast-slow economic spectrum, in line with our hypothesis. Although a few studies have pointed out the decoupling evolution and ecological adaptation between leaf and stem (Baraloto et al., 2010; Fortunel et al., 2012), the resulting coordinated variations between leaf and stem traits supports the findings of other studies (Chave et al., 2009; Freschet et al., 2010; Poorter et al., 2010; Fortunel et al., 2012; Reich and Cornelissen, 2014; de la Riva et al., 2016) and provides new evidence of the LES and SES in tropical cloud forests (Figure 1). This finding contributes to better understanding of the ecological trade-off for species in high-altitude tropical forests.

Specifically, we found that SLA, LN, and LP were positively correlated with each other but negatively correlated with LMA, LT, and WD. Such a coordinated trait variations reflects an ecological trade-off between acquisitive and conservative resource utilizations. With higher SLA, LN, and LP, the resource-acquisitive species (such as *Schefflera octophylla* and *Polyalthia plagioneura*) are characterized by strong photosynthetic capacity, short lifespan, and high growth rates. By contrast, the resource-conservative species with higher LMA, LT, and WD (such as *Syzygium buxifolium*, *Polyspora axillaris*, and *Osmanthus hainanensis*) are characterized by weak photosynthetic capacity, long lifespan and low growth rate. Moreover, the WD was significantly correlated with LMA, SLA, LT, with the SMA slopes at log scale significantly different from +1 or −1. Specifically, the SMA slopes that remarkably deviate from +1.0 or −1.0 (i.e., the scaling relationships are disproportionate at log scale) reflect that resources are disproportionately allocated within plants according to specific plant functions (Long et al., 2020). Therefore, our results indicate not only the ecological trade-off but also the allometric allocation.

These patterns may be attributed to the pronounced environmental stress in the tropical cloud forest. For examples, for withstanding the frequent and strong winds, some species have to adjust the toughness (or persistence) of leaf and stem in a coordinated way and tend to have more investments on stem construction to increase the persistence of the whole plants (Zheng and Martínez-Cabrera, 2013). When the micro-environment is stressful, some species have to be resource-conservative and resistant. For example, canopy species or species distributed at windward slopes should be adapted to stronger winds (Long et al., 2011b). These trees can reinforce their resistance by e.g., elevating tissue density and toughness, at the cost of reducing the resource input for photosynthesis and respiration (Coley and Barone, 1996; Agrawal and Fishbein, 2006; Hanley et al., 2007). On the contrary, in the face of the less stressful conditions, some species can be resource-acquisitive, with rapid resource acquisition and high growth rate (Wright and Westoby, 2002). These species often increase the SLA to facilitate photosynthesis and reduce the WD to accelerate material transports. Such a strategic trade-off may arise from species’ adaptation to local environments of the tropical cloud forest such as strong winds, low temperatures, clouds and fog, and intensive radiation.

### The Multi-Dimensional Resource Acquisition Strategies in Roots

In contrast to the root economics spectrum (RES) (Reich and Cornelissen, 2014), we found that SRL and RTD poorly correlated with RN and RP (Table 2 and Figure 2), indicating that the trade-off among fine root traits of tropical cloud forest trees is not one-dimensional. Specifically, as displayed in the PCA (Figure 1), variations of RN and RP (represented by the first axis) are correlated but nearly orthogonal with those of SRA, SRL and RTD (represented by the second axis). These results are inconsistent with the RES of temperate tree species, herbaceous plants, and some tropical rain forest species (Reich et al., 2008; Makita et al., 2012).

SRL, SRA, RN, RP are all related to plants nutrient uptakes. For examples, SRA and SRL, respectively indicate the absorption area per unit mass and the absorption length per unit mass of the fine root, and nutrient absorption of the fine roots will be facilitated by larger SRA and SRL. RN and RP reflect the resource acquisition ability and metabolic activity, partly because higher N and P contents contribute to the load of root phloem (Tjoelker et al., 2005; Kerkhoff et al., 2006; Kong et al., 2016). Therefore, it is expected that these traits are positively correlated, as illustrated by plant RES (Reich and Cornelissen, 2014). However, our results demonstrate that changes of root nutrient traits were decoupled from those of root morphological traits, which may be due to their differentiations in function and susceptibility (to environmental conditions).

Even if both root nutrient and morphological traits essentially matter to plant nutrient absorption (they act through physiological and physical processes, respectively), it should be noted that the latter also relate to plant defense mechanism (Santiago et al., 2004; Wright et al., 2005; Poorter and Bongers, 2006; Baraloto et al., 2010; Wilson, 2014). For example, in the face of strong winds, trees with well-developed roots can solidly grow in the ground. More importantly, root nutrient traits are more sensitive to soil chemical properties such as acidity and fertility (Comas et al., 2012), while root morphological traits are more susceptible to soil physical properties such as soil texture and thickness (Clark et al., 2003; Bejarano et al., 2010; Alameda and Villar, 2012). In other words, traits in response to different stress factors may vary independently. Note that these may be especially the case in the tropical cloud forest studied here. Specifically, this forest, located in the high-altitude ridges and mountaintops, is notoriously characterized by the pronounced environmental stresses caused by strong winds, soil phosphorus deficiency and thin soil layers (Long et al., 2011a).

Moreover, roots face a more complex trade-off of resource utilization in consideration of the complexity of soil nutrients. For example, the acquisition of a mobile nutrient such as nitrate can be optimized by enhancing SRL or the capacity to proliferate in resource-rich patches, whereas immobile nutrients such as P may require high root hair density, prolific root branching, or mycorrhizal symbiosis (Comas et al., 2012). This implies that traits considered acquisitive for the uptake of one particular resource are not necessarily acquisitive for the uptake of another. So the simultaneous uptake of different resources may be optimized by different traits, depending on the most limiting resource. In the BWL tropical cloud forest where soil P is the most limiting resource (Long et al., 2011a), adaptation mechanisms of trees may be multiple or depend on other traits such as the density of root hairs and the number of adventitious roots (Lambers et al., 2006; Lynch and Brown, 2008), so that changes of the root nutrient and traits studied here are decoupled.

Our research shows that in the tropical cloud forest, unlike the leaf traits with a one-dimensional LES, the fine root traits of trees do not have a one-dimensional PES, but develop a resource acquisition strategy with independent trade-offs within morphological traits and nutrient traits. These findings support the multi-dimensional hypothesis of fine root traits, indicating that multi-dimensional relationships among root traits enable plants to better adapt and explain the variation of root traits in different habitats, scales, and species (Weemstra et al., 2016). The resource acquisition of fine roots is complex and diverse. The traits related to resource acquisition are not limited to morphological traits such as SRL and RTD. Future research should also include root density, hair root length and density, branch strength, and anatomical traits (e.g., cortical thickness) in fine root traits to better understand underground resource acquisition strategies of the tropical cloud forests.

### Coordinated Patterns for Nutrient Traits but Decoupled Patterns for Morphological Traits

We found that RN and RP were significantly positively correlated with LN, LP, WN, and WP in tropical cloud forests (Figure 2). These findings are consistent with the findings of Aekerly and Donoghue (1998), Craine et al. (2005), Tjoelker et al. (2005), Kerkhoff et al. (2006), and Wang et al. (2017), supporting the overall coordination hypothesis (Mommer and Weemstra, 2012). Such a coordinated nutrient trait change reflects that the nutrient transports between the above- and below-ground parts follow a generally consistent rule across species, and that the above- and belowground ecological processes related to nutrient cycling are closely correlated (Zhao et al., 2016). Plant N and P contents matter to intrinsic physiology (Kerkhoff et al., 2006; Valverde-Barrantes et al., 2015). As the fundamental nutrient elements mattering to cell metabolisms, N and P should be systematically allocated within plants, allowing plant physiological processes to be optimized and cooperatively accomplished by above- and below-ground organs.

In line with other studies (Craine et al., 2005; Tjoelker et al., 2005; Baraloto et al., 2010; Fortunel et al., 2012), our study revealed that the morphological traits of roots hardly correlate with those of stems and leaves (Table 2), which does not support the functional similarity hypothesis (Baraloto et al., 2010; Wang et al., 2017). The decoupling of aboveground and underground morphological traits may be due to the effects of multiple concurrent process. Plants may be susceptible to multiple processes or factors (Bubb et al., 2004; Long et al., 2011b; Bardgett et al., 2014; Valverde-Barrantes et al., 2015; Weemstra et al., 2016), so that they are likely to separately adjust the functional traits of different organs (Freschet et al., 2013; Laughlin, 2014). In particular, for trees in the tropical cloud forests, their above-ground morphological traits may be more susceptible to microclimate, but their below-ground morphological traits may be more sensitive to soil conditions. Moreover, Valverde-Barrantes et al. (2015) and Wang et al. (2017) found that the morphological traits of roots have stronger phylogenetic conservation trends than those of leaves, which may also lead to such a decoupling of the morphological traits between above-and below-ground parts.

## Conclusion

**FIGURE 4:**
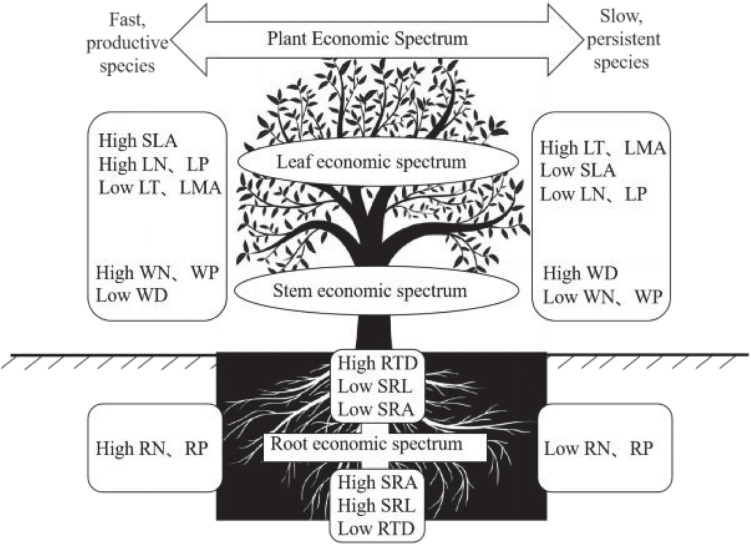
Conceptual illustration of the plant economic spectrum (PES). The nutrient traits of fine roots, such as RN and RP, are consistent with the leaf economic spectrum (LES), while the morphological traits of fine roots, such as RTD, SRL, and SRA, are independent of the PES.

In conclusion, in the BWL tropical cloud forest, we found a PES characterized by leaf and stem, which reflects a trade-off between acquisitive and conservative resource utilization (Figure 4). Such a pronounced ecological trade-off may be caused by the strong environmental stress to which species have to adapt locally. We also found that changes of root traits are multidimensional, and that only the changes of nutrient traits are coordinated between plant above- and below-ground parts Figure 4. These outcomes suggest that plants in this forest may respond to local environmental conditions in a complex way through developing several strategies related to different traits or organs. This may stem from the fact that the environmental stress in this forest is related to multiple factor. As a whole, by taking the morphological and nutrient traits of root, stem and leaf into consideration, our findings contribute to a comprehensive understanding of the PES and ecological trade-off in the tropical cloud forest community, potentially providing a key insight into the species coexistence and community assembly in this forest.

## Data Availability Statement

The raw data supporting the conclusions of this article will be made available by the authors, without undue reservation, to any qualified researcher.

## Author Contributions

YY and WL conceived the project, performed the data analyses, and wrote the manuscript. YY, CX, XW, and GL designed and performed all the field work and laboratory experiments. GF provided some suggestions and assistance for writing this manuscript.

## Conflict of Interest

The authors declare that the research was conducted in the absence of any commercial or financial relationships that could be construed as a potential conflict of interest.

## References

[B1] Aekerly D. D. Donoghue M. J. (1998). Leaf size, sapling allometry, and corner’s rules: phylogeny and correlated evolution in maples (acer). *Am. Nat.* 152 767–791. 10.1086/286208 18811427

[B2] Agrawal A. A. Fishbein M. (2006). Plant defense syndromes. *Ecology* 87 S132–S149. 16922309 10.1890/0012-9658(2006)87[132:pds]2.0.co;2

[B3] Alameda D. Villar R. (2012). Linking root traits to plant physiology and growth in Fraxinus angustifolia Vahl. seedlings under soil compaction conditions. *Environ. Exp. Bot.* 79 49–57. 10.1016/j.envexpbot.2012.01.004

[B4] Baraloto C. Paine C. E. T. Poorter L. Beauchene J. Bonal D. Domenach A. M. (2010). Decoupled leaf and stem economics in rain forest trees. *Ecol. Lett.* 13 1338–1347. 10.1111/j.1461-0248.2010.01517.x 20807232

[B5] Bardgett R. D. Mommer L. Vries F. T. D. (2014). Going underground: root traits as drivers of ecosystem processes. *Trends Ecol. Evol.* 29 692–699. 10.1016/j.tree.2014.10.006 25459399

[B6] Bejarano M. D. Villar R. Murillo A. M. Quero J. L. (2010). Effects of soil compaction and light on growth of *Quercus pyrenaica* Willd. (Fagaceae) seedlings. *Soil Tillage Res.* 110 108–114. 10.1016/j.still.2010.07.008

[B7] Bu W. S. Zang R. G. Ding Y. (2014). Field observed relationship between biodiversity and ecosystem functioning during secondary succession in a tropical lowland rainforest. *Acta Oecol.* 55 1–7. 10.1016/j.actao.2013.10.002

[B8] Bubb P. May I. Miles L. Sayer J. (2004). *Cloud Forest Agenda.* Cambridge: UNEP-WCMC.

[B9] Chapin F. S. (1980). The mineral nutrition of wild plants. *Annu. Rev. Ecol. Syst.* 11 233–260. 10.1146/annurev.es.11.110180.001313

[B10] Chave J. Coomes D. Jansen S. Lewis S. L. Swenson N. G. Zanne A. E. (2009). Towards a worldwide wood economics spectrum. *Ecol. Lett.* 12 351–366. 10.1111/j.1461-0248.2009.01285.x 19243406

[B11] Clark L. J. Whalley W. R. Barraclough P. B. (2003). How do roots penetrate strong soil? *Plant Soil* 255 93–104. 10.1023/a:1026140122848

[B12] Coley P. D. Barone J. A. (1996). Herbivory and plant defenses in tropical forests. *Annu. Rev. Ecol. Syst.* 27 305–335. 10.1146/annurev.ecolsys.27.1.305

[B13] Comas L. H. Mueller K. E. Taylor L. L. Midford P. E. Callahan H. S. Beerling D. J. (2012). Evolutionary patterns and biogeochemical significance of angiosperm root traits. *Int. J. Plant Sci.* 173 584–595. 10.1086/665823

[B14] Cornelissen J. H. C. Lavorel S. Garnier E. Díaz S. Buchmann N. Gurvich D. E. (2003). A handbook of protocols for standardized and easy measurement of plant functional traits worldwide. *Aust. J. Bot.* 51 335–380. 10.1071/bt02124

[B15] Cornwell W. K. Schwilk D. W. Ackerly D. D. (2006). A trait based test for habitat filtering: convex hull volume. *Ecology* 87 1465–1471. 10.1890/0012-9658 16869422

[B16] Cornwell W. K. Ackerly D. D. (2009). Community assembly and shifts in the distribution of functional trait values across an environmental gradient in coastal California. *Ecol. Monogr.* 79 109–126. 10.1890/07-1134.1

[B17] Craine J. Lee W. Bond W. (2005). Environmental constraints on a global relationship among leaf and root traits of grasses. *Ecology* 86 12–19. 10.1890/04-1075

[B18] de la Riva E. G. Tosto A. Pérez-Ramos I. M. Navarro-Fernández C. M. Olmo M. Anten N. P. (2016). A plant economics spectrum in Mediterranean forests along environmental gradients: is there coordination among leaf, stem and root traits? *J. Veg. Sci.* 27 187–199. 10.1111/jvs.12341

[B19] Diaz S. Hodgson J. G. Thompson K. Cabido M. Cornelissen J. H. C. Jalili A. (2004). The plant traits that drive ecosystems: evidence from three continents. *J. Veg. Sci.* 15 295–304.

[B20] Enquist B. J. West G. B. Charnov E. L. Brown J. H. (1999). Allometric scaling of production and life-history variation in vascular plants. *Nature* 401 907–911. 10.1038/44819

[B21] Fortunel C. Fine P. V. A. Baraloto C. (2012). Leaf, stem and root tissue strategies across 758 neotropical tree species. *Funct. Ecol.* 26 1153–1161. 10.1111/j.1365-2435.2012.02020.x

[B22] Freschet G. T. Bellingham P. J. Lyver P. O. Bonner K. I. Wardle D. A. (2013). Plasticity in above- and belowground resource acquisition traits in response to single and multiple environmental factors in three tree species. *Ecol. Evol.* 3 1065–1078. 10.1002/ece3.520 23610644 PMC3631414

[B23] Freschet G. T. Cornelissen J. H. C. van Logtestijn R. S. P. Aerts R. (2010). Evidence of the ‘plant economics spectrum’ in a subarctic flora. *J. Ecol.* 98 362–373. 10.1111/j.1365-2745.2009.01615.x

[B24] Garnier E. Navas M. L. (2012). A trait-based approach to comparative functional plant ecology: concepts, methods and applications for agroecology. *Agron. Sustain. Dev.* 32 365–399. 10.1007/s13593-011-0036-y

[B25] Grime J. P. (1977). Evidence for the existence of three primary strategies in plants and its relevance to ecological and evolutionary theory. *Am. Nat.* 111 1169–1194. 10.1086/283244

[B26] Guo D. Xia M. Wei X. Chang W. Liu Y. Wang Z. (2008). Anatomical traits associated with absorption and mycorrhizal colonization are linked to root branch order in twenty-three chinese temperate tree species. *New Phytol.* 180 673–683. 10.1111/j.1469-8137.2008.02573.x 18657210

[B27] Hanley M. E. Lamont B. B. Fairbanks M. M. Rafferty C. M. (2007). Plant structural traits and their role in anti-herbivore defence. *Perspect. Plant Ecol. Evol. Syst.* 8 157–178. 10.1016/j.ppees.2007.01.001

[B28] Kerkhoff A. J. Fagan W. F. Elser J. J. Enquist B. J. (2006). Phylogenetic and growth form variation in the scaling of nitrogen and phosphorus in the seed plants. *Am. Nat.* 168 E103–E122. 17004214 10.1086/507879

[B29] Kong D. L. Wang J. J. Kardol P. Wu H. F. J. Zeng H. Deng X. B. (2016). Economic strategies of plant absorptive roots vary with root diameter. *Biogeosciences* 13 415–424. 10.5194/bg-13-415-2016

[B30] Kramer-Walter K. R. Bellingham P. J. TMillar R. Smissen R. D. Richardson S. J. Laughlin D. C. (2016). Root traits are multidimensional: specific root length is independent from root tissue density and the plant economic spectrum. *J. Ecol.* 104 1299–1310. 10.1111/1365-2745.12562

[B31] Lambers H. Shane M. W. Cramer M. D. Pearse S. J. Veneklaas E. J. (2006). Root structure and functioning for efficient acquisition of phosphorus: matching morphological and physiological traits. *Ann. Bot.* 98 693–713. 10.1093/aob/mcl114 16769731 PMC2806175

[B32] Laughlin D. C. (2014). The intrinsic dimensionality of plant traits and its relevance to community assembly. *J. Ecol.* 102 186–193. 10.1111/1365-2745.12187

[B33] Long W. X. Zang R. G. Ding Y. (2011a). Air temperature and soil phosphorus availability correlate with trait differences between two types of tropical cloud forests. *Flora* 206 896–903. 10.1016/j.flora.2011.05.007

[B34] Long W. X. Zang R. G. Schamp B. S. Ding Y. (2011b). Within- and among-species variation in specific leaf area drive community assembly in a tropical cloud forest. *Oecologia* 167 1103–1113. 10.1007/s00442-011-2050-9 21695546

[B35] Long W. X. Zhou Y. D. Schamp B. S. Zang R. G. Yang X. B. Poorter L. (2020). Scaling relationships among functional traits are similar across individuals, species, and communities. *J. Veg. Sci.* 31 571–580. 10.1111/jvs.12893

[B36] Lynch J. P. Brown K. M. (2008). “Root strategies for phosphorus acquisition,” in *The Ecophysiology Of Plant–Phosphorus Interactions*, eds White P. J. Hammond J. P. (Dordrecht: Springer), 83–116. 10.1007/978-1-4020-8435-5_5

[B37] Makita N. Kosugi Y. Dannoura M. Takanashi S. Niiyama K. Kassim A. R. (2012). Patterns of root respiration rates and morphological traits in 13 tree species in a tropical forest. *Tree Physiol.* 32 303–312. 10.1093/treephys/tps008 22367761

[B38] Mommer L. Weemstra M. (2012). The role of roots in the resource economics spectrum. *New Phytol.* 195 725–727. 10.1111/j.1469-8137.2012.04247.x 22861183

[B39] Poorter H. Niklas K. J. Reich P. B. Oleksyn J. Poot P. Mommer L. (2012). Biomass allocation to leaves, stems and roots: meta-analyses of interspecific variation and environmental control. *New Phytol.* 193 30–50. 10.1111/j.1469-8137.2011.03952.x 22085245

[B40] Poorter L. Bongers F. (2006). Leaf traits are good predictors of plant performance across 53 rain forest species. *Ecology* 87 1733–1743. 10.1890/0012-9658(2006)87[1733:ltagpo]2.0.co;2 16922323

[B41] Poorter L. Mcdonald I. Alarcón A. Fichtler E. Licona J. C. Peña-Claros M. (2010). The importance of wood traits and hydraulic conductance for the performance and life history strategies of 42 rainforest tree species. *New Phytol.* 185 481–492. 10.1111/j.1469-8137.2009.03092.x 19925555

[B42] Poorter L. Wright S. J. Paz H. Ackerly D. D. Condit R. Ibarra-Manriques G. (2008). Are functional traits good predictors of demographic rates? *Evid. Five Neotrop. For. Ecol.* 89 1908–1920. 10.1890/07-0207.1 18705377

[B43] Pratt R. B. Jacobsen A. L. Ewers F. W. Davis S. D. (2007). Relationships among xylem transport, biomechanics and storage in stems and roots of nine *Rhamnaceae* species of the California chaparral. *New Phytol.* 174 787–798. 10.1111/j.1469-8137.2007.02061.x 17504462

[B44] Prieto I. Roumet C. Cardinael R. Dupraz C. Jourdan C. Kim J. H. (2015). Root functional parameters along a land-use gradient: evidence of a community-level economics spectrum. *J. Ecol.* 103 361–373. 10.1111/1365-2745.12351

[B45] Reich P. B. Cornelissen H. (2014). The world–wide ‘fast–slow’ plant economics spectrum: a traits manifesto. *J. Ecol.* 102 275–301. 10.1111/1365-2745.12211

[B46] Reich P. B. Tjoelker M. G. Walters M. B. Vanderklein D. W. Bushena C. (1998). Close association of RGR, leaf and root morphology, seed mass and shade tolerance in seedlings of nine boreal tree species grown in high and low light. *Funct. Ecol.* 12 327–338. 10.1046/j.1365-2435.1998.00208.x

[B47] Reich P. B. Walters M. B. Ellsworth D. S. (1997). From tropics to tundra: global convergence in plant functioning. *Proc. Natl. Acad. Sci. U.S.A.* 94 13730–13734. 10.1073/pnas.94.25.13730 9391094 PMC28374

[B48] Reich P. B. Tjoelker M. G. Pregitzer K. S. Wright I. J. Oleksyn J. Machado J. L. (2008). Scaling of respiration to nitrogen in leaves, stems and roots of higher land plants. *Ecol. Lett.* 11 793–801. 18445031 10.1111/j.1461-0248.2008.01185.x

[B49] Ryser P. Eek L. (2000). Consequences of phenotypic plasticity vs. interspecific differences in leafand root traits for acquisition of aboveground and belowground resources. *Am. J. Bot.* 87 402–411. 10.2307/2656636 10719001

[B50] Santiago L. S. Goldstein G. Meinzer F. C. Fisher J. B. Machado K. Woodruff D. (2004). Leaf photosynthetic traits scale with hydraulic conductivity and wood density in Panamanian forest canopy trees. *Oecologia* 140 543–550. 10.1007/s00442-004-1624-1 15232729

[B51] Stadtmüller T. (1987). *Cloud Forest In The Humid Tropics: A Bibliographic Review.* Tokyo: United Nations University Press.

[B52] Tjoelker M. G. Craine J. M. Wedin D. Reich P. B. Tilman D. (2005). Linking leaf and root trait syndromes among 39 grassland and savannah species. *New Phytol.* 167 493–508. 10.1111/j.1469-8137.2005.01428.x 15998401

[B53] Tyree M. T. Ewers F. W. (1991). The hydraulic architecture of trees and other woody plants. *New Phytol.* 119 345–360. 10.1111/j.1469-8137.1991.tb00035.x

[B54] Valverde-Barrantes O. J. Smemo K. A. Blackwood C. B. (2015). Fine root morphology is phylogenetically structured, but nitrogen is related to the plant economics spectrum in temperate trees. *Funct. Ecol.* 29 796–807. 10.1111/1365-2435.12384

[B55] Wang R. L. Wang Q. F. Zhao N. Yu G. R. He N. P. (2017). Complex trait relationships between leaves and absorptive roots: coordination in tissue n concentration but divergence in morphology. *Ecol. Evol.* 7 2697–2705. 10.1002/ece3.2895 28428860 PMC5395436

[B56] Warton D. I. Wright I. J. Falster D. S. Westoby M. (2006). Bivariate line-fitting methods for allometry. *Biol. Rev.* 81 259–291. 10.1017/s1464793106007007 16573844

[B57] Weemstra M. Mommer L. Visser E. J. van Ruijveny J. Kuyper T. W. Mohren G. M. (2016). Towards a multidimensional root trait framework: a tree root review. *New Phytol.* 211 1159–1169. 10.1111/nph.14003 27174359

[B58] Westoby M. (1998). A leaf-height-seed (LHS) plant ecology strategy scheme. *Plant Soil* 199 213–227.

[B59] Westoby M. Wright I. J. (2006). Land-plant ecology on the basis of functional traits. *Trends Ecol. Evol.* 21 261–268. 10.1016/j.tree.2006.02.004 16697912

[B60] Westoby M. Falster D. S. Moles A. T. Vesk P. A. Wright I. J. (2002). Plant ecological strategies: some leading dimensions of variation between species. *Annu. Rev. Ecol. Syst.* 33 125–159. 10.1146/annurev.ecolsys.33.010802.150452

[B61] Wilson S. D. (2014). Below-ground opportunities in vegetation science. *J. Veg. Sci.* 25 1117–1125. 10.1111/jvs.12168

[B62] Wright I. J. Westoby M. (2002). Leaves at low versus high rainfall: coordination of structure, lifespan and physiology. *New Phytol.* 155 403–416. 10.1046/j.1469-8137.2002.00479.x 33873314

[B63] Wright I. J. Reich P. B. Cornelissen J. H. C. Falster D. S. Groom P. K. Hikosaka K. (2005). Modulation of leaf economic traits and trait relationships by climate. *Glob. Ecol. Biogeogr.* 14 411–421. 10.1111/j.1466-822x.2005.00172.x

[B64] Wright I. J. Reich P. B. Westoby M. Ackerly D. D. Baruch Z. Bongers F. (2004). The worldwide leaf economics spectrum. *Nature* 428 821–827. 15103368 10.1038/nature02403

[B65] Zhao N. Yu G. R. He N. P. Wang Q. F. Guo D. L. Zhang X. Y. (2016). Coordinated pattern of multi-element variability in leaves and roots across chinese forest biomes. *Glob. Ecol. Biogeogr.* 25 359–367. 10.1111/geb.12427

[B66] Zheng J. M. Martínez-Cabrera H. I. (2013). Wood anatomical correlates with the theoretical conductivity and wood density across China: evolutionary evidence of the functional differentiation of axial and radial parenchyma. *Ann. Bot.* 112 927–935. 10.1093/aob/mct153 23904446 PMC3747806

